# How Can the Operating Environment for Nutrition Research Be Improved in Sub-Saharan Africa? The Views of African Researchers

**DOI:** 10.1371/journal.pone.0066355

**Published:** 2013-06-12

**Authors:** Kathleen Van Royen, Carl Lachat, Michelle Holdsworth, Karlien Smit, Joyce Kinabo, Dominique Roberfroid, Eunice Nago, Christopher Garimoi Orach, Patrick Kolsteren

**Affiliations:** 1 Department of Food Safety and Food Quality, Ghent University, Ghent, Belgium; 2 Unit of Nutrition and Child Health, Institute for Tropical Medicine, Antwerp, Belgium; 3 ScHARR- Section of Public Health, University of Sheffield, Sheffield, United Kingdom; 4 Africa Unit for Transdisciplinary Health Research, North-West University, Potchefstroom, South Africa; 5 Department of Food Science and Technology, Sokoine University, Morogoro, Tanzania; 6 Faculty of Agricultural Sciences, University of Abomey-Calavi, Abomey-Calavi, Benin; 7 School of Public Health, Makerere University, Kampala, Uganda; Consejo Superior de Investigaciones Cientifics, Spain

## Abstract

Optimal nutrition is critical for human development and economic growth. Sub-Saharan Africa is facing high levels of food insecurity and only few sub-Saharan African countries are on track to eradicate extreme poverty and hunger by 2015. Effective research capacity is crucial for addressing emerging challenges and designing appropriate mitigation strategies in sub-Saharan Africa. A clear understanding of the operating environment for nutrition research in sub-Saharan Africa is a much needed prerequisite. We collected data on the barriers and requirements for conducting nutrition research in sub-Saharan Africa through semi-structured interviews with 144 participants involved in nutrition research in 35 countries in sub-Saharan Africa. A total of 133 interviews were retained for coding. The main barriers identified for effective nutrition research were the lack of funding due to poor recognition by policymakers of the importance of nutrition research and under-utilisation of research findings for developing policy, as well as an absence of research priority setting from within Africa. Current research topics were perceived to be mainly determined by funding bodies from outside Africa. Nutrition researchers argued for more commitment from policymakers at national level. The low capacity for nutrition research was mainly seen as a consequence of insufficient numbers of nutrition researchers, limited skills and a poor research infrastructure. In conclusion, African nutrition researchers argued how research priorities need to be identified by African stakeholders, accompanied by consensus building to enable creating a problem-driven national research agenda. In addition, it was considered necessary to promote interactions among researchers, and between researchers and policymakers. Multidisciplinary research and international and cross-African collaboration were seen as crucial to build capacity in sub-Saharan nutrition research.

## Introduction

Sub-Saharan Africa is facing persisting high levels of food insecurity and malnutrition [1]. Although the region has benefited from economic growth, food security for a vast proportion of the African population is still precarious [2]. Whereas 14% of the global population is estimated to be undernourished, this prevalence is about 33% in sub-Saharan Africa [3]. As undernutrition affects cognitive development, educational outcomes, work capacity and gross domestic product [4], improving nutrition is a priority and essential for both human development and economic growth of the continent [2], 5. In addition, over the last decades the prevalence of overweight and obesity has increased in many urban and some rural parts of sub-Saharan Africa and is a rapidly growing threat to public health and development in the region [6]. The World Health Organization (WHO) recognised this in its Global Strategy on Diet, Physical Activity and Health [7] and a more recent action plan focussing on preventing non-communicable diseases worldwide, including sub-Saharan Africa [8]. Recently, there has been renewed attention for nutrition. The Scaling Up Nutrition (SUN) [9] movement aims to mobilise a wide range of stakeholders to fight hunger and undernutrition. New funding schemes such as the Bill and Melinda Gates Foundation [10], the Global Alliance of Improved Nutrition (GAIN) [11], the Department for International Development (DFID) [12] and the New Alliance for Food security and Nutrition [13] dedicate substantial funds to improve nutrition in Africa.

Despite this, only few countries in sub-Saharan Africa are on track to achieve the UN Millennium Development Goals to eradicate extreme poverty and hunger by 2015 [14], [15]. In addition to persistent political, socio-economic and technological challenges, emerging environmental threats such as climate change, new diseases, urbanisation, migration, water and land availability, as well as globalisation, are likely to have a profound impact on nutrition in Africa [2].

Nutrition research from Africa is critical as it allows informed action towards what works best on the continent. However, nutrition research output from sub-Saharan Africa is scarce and provides insufficient evidence for applied solution-based action [16]. Adequate research capacity is crucial for addressing emerging challenges and designing appropriate mitigation strategies in sub-Saharan Africa [17]. Empowering the research environment for nutrition is therefore one of the precursors to economic development in sub-Saharan Africa. A better understanding of specific barriers, drivers and unmet needs perceived by nutrition researchers can help build capacity and prioritise investment in nutrition research in the region.

This article presents an assessment of the perceptions regarding the operating environment for nutrition research in sub-Saharan Africa of a large sample of African nutrition researchers. This study was carried out as part of the project, called SUNRAY “Sustainable Nutrition Research for Africa in the Years to come” (www.sunrayafrica.co.za). SUNRAY aims to facilitate sustainable nutrition research in Africa by developing a strategic framework for researchers, decision makers and other stakeholders working to improve the nutrition situation in Africa.

## Methods

Data were collected by semi-structured interviews with people actively involved in nutrition research in sub-Saharan Africa, either as academics or as a member of an international organisation, Non-Governmental Organisation or a public body (called nutrition researchers hereafter). A convenience sample of respondents was contacted through existing networks of the SUNRAY partners such as the Federation of African Nutrition Societies (FANUS) and the African Nutrition Leadership Program (ANLP). Respondents were recruited by using a snowball method, i.e. referral from primary recruits of the networks to other relevant respondents. The aim was to include at least 5 interviews in each of the 47 countries of sub-Saharan Africa [18], making a target of 235 interviews. Mayotte was not included because it is part of France.

In total 144 interviews were carried out, with researchers in 35 different countries, between August 2011 and March 2012, using a semi-structured telephone or Voice over Internet Protocol administered interview (n = 104). Other interviews (n = 17) were conducted as self-completion by participants using a hardcopy of the questionnaire, due to limited internet or telephone connectivity. Some interviews (n = 23) were conducted face to face, when this was preferred. Interviews could not be conducted in 12 of the 48 countries (Angola, Comoros, Equatorial Guinea, Eritrea, Malawi, Mauritius, Rwanda, Sudan -North and South-, São Tomé and Príncipe, Seychelles, Sierra Leone and Somalia). We were unable to identify or contact eligible respondents in these countries or there was no response received from potential participants.

To ensure uniform data collection and maximise comparability, an instruction guide with information on both technical and methodological aspects was developed for the interviewers. This guide provided practical standard recommendations for obtaining informed consent, as well as conducting (probing questions), recording and transcribing the interviews. Interviews were conducted by trained researchers affiliated to the SUNRAY partner universities from Belgium, Benin, South-Africa, Tanzania and Uganda. Each interviewer conducted interviews in the respective region in sub-Saharan Africa countries where the interviewer's language was spoken. Interviews were carried out in English, French, Portuguese or Afrikaans, depending on the language spoken by the respondent.

The interview assessed the perceptions of drivers and constraints for conducting nutrition research from the perspective of researchers in sub-Saharan Africa. In addition, suggestions for improvement and future research priorities were collected. A semi-structured questioning route was developed, since no suitable or previously validated instrument was available. Interview questions were derived primarily from experiences of the project partners and based on themes emerging in reports on research capacity assessments [19], [20]. Open questions such as ‘Is nutrition research seen as a priority in your country?’ were included, followed by specific probing questions like ‘what should be done to make it a priority?’ in case of a negative answer. The questioning route was revised several times by the SUNRAY consortium partners and modified based on the suggestions from respondents in the sample where the survey was pretested. The interview was pre-tested on a convenience sample of (n = 6) active nutrition researchers in sub-Saharan Africa (in Ethiopia and Uganda), not included in the final sample, to assess content and face validity.

All interviews were recorded using voice recorders and transcribed verbatim by researchers for further data analysis. Interview transcripts in English and French were directly used for coding. Transcripts in Afrikaans and Portuguese were translated into English and verified by a second researcher. Data were analysed using basic content analysis [21]. The software Epidata (Odense, Denmark) was used to enter codes for the data. One researcher read the interview transcripts, coded the answers into recurring themes, developed a codebook and finally allocated new codes for emerging themes. Afterwards, themes were grouped into categories of similar meaning. In case of any doubt in the coding and recoding process, a second researcher was consulted until consensus was reached. To provide an overview of the most reported responses, a content analysis was conducted by calculating the frequencies for each of the themes in Stata 9 (Statacorps, Texas, USA).

Interviews were included if they concerned (i) researchers currently conducting nutrition research, (ii) researchers not conducting research but having at least 5 years of nutrition research experience (iii) researchers currently applying for funds for nutrition research, and (iv) interviews with an adequate audio recording quality. For the analysis, 11 interviews were excluded because the characteristics of these interviews did not meet the inclusion criteria.

Results in this report are presented as semi-quantitative data with percentages of emerging topics from the answers. For open ended questions or questions with multiple answers, various themes could be extracted per interviewee. Tables therefore show the themes and how frequently they occurred (n) in the answers, with corresponding percentages based on the total number of answers. To avoid confusion on the unit of analysis, we refer to ‘% of responses’ where needed. Qualitative excerpts from the interviews are provided for a more in-depth understanding of the issues presented by the interviewees where appropriate.

This study received ethical approval from the Institutional Review Board of the Institute of Tropical Medicine, Belgium on June 8, 2011 (nr 11 21 3 771) and the Higher Degrees, Research and Ethics Committee of Makerere University, Uganda on July 22, 2011 (nr 137). The first institute was responsible for the overall coordination of the SUNRAY project and the latter for the data collection of the researcher interviews specifically. All participants provided written informed consent. In case written informed consent could not be provided, verbal consent (through audio-recording) was obtained. In some cases written consent could not be obtained due to limited technology access (fax, scanner, internet).

## Results

### Participants

The responses of a final sample of 133 interviews were analysed. Their characteristics are presented in Table 1. All participants were aged between 24 to 68 years (mean age: 42.9±0.8 years) and were active nutrition researchers in sub-Saharan Africa. The mean duration of research experience was 12.4±1.0 years.

**Table 1:** Descriptive characteristics of the study sample.

Participants	n	%^a^
Total	133	100
Male	66	50
**Highest degree obtained**		
PhD	54	41
Master	56	42
Bachelor	7	5
Other	14	10
**Current institution** b		
Academic	68	49
Public	39	28
NGO	13	9
Other	19	13

a *Missing responses are not tabulated.*

b *Only the first institution of affiliation is reported.*

### Making nutrition research a priority

More than half (61%) of the interviewees reported that nutrition research was not considered to be a priority in their country (Table 2). The most frequent reasons given for this were: (i) the limited commitment of the government to nutrition research, in particular a lack of financial support (21% of responses) and (ii) a lack of attention given to nutrition research (22% of responses). Some of these researchers attributed the poor attention to governments prioritising reactive and emergency nutrition interventions. Another significant constraint here was the perception that there was low capacity to conduct nutrition research (16% of responses). Funding and interest from either governments or donors were often primarily centred on other health issues or on curative aspects of nutrition research rather than prevention (11% of responses).

**Table 2:** Nutrition research as a national priority as perceived by nutrition researchers in sub-Saharan Africa.

Is nutrition research a national priority?	n = 133^a^	%
No	82	61
Yes, but not enough (due to constraints)	26	20
It has not been a priority, but now it is becoming one	6	4
Yes	18	13

Reasons for nutrition research not being a national priority	n = 185^b^	%
No attention from government, i.e. limited awareness and understanding, reactive approach	40	22
No funding from government for research or follow-up	39	21
Low capacity to do nutrition research	29	16
Priority/funds go to health in general/no specific nutrition donors	20	11
No coordinating body at governmental level	17	9
Research themes are donor or government dependent	16	9
Nutrition research done for personal or donor interest/for degrees	12	6
Priority-setting and discourse, but no action by government	9	5
Many other priorities due to context of conflict before or currently	3	2
No input of nutrition researchers at national level	2	1

Suggestions to make nutrition research a national priority	n = 200^b^	%
More financial support from government	38	19
National implemented priority agenda	37	18
Need for more capacity to do research (skills/equipment)	26	13
Attention from government	24	12
Advocacy from nutrition researchers for nutrition research	23	11
Coordinating body or centre for nutrition research	13	7
Address other research methods or topics, according to current problems	8	4
Collaboration organisations/researchers	8	4
More interest from donors for nutrition agenda/national agenda	6	3
Education/awareness on nutrition for general population	7	4
Need for a national supportive nutrition policy	4	2
Others	6	3

a *Number of researchers as only one answer was possible for this question.*

b *Total number of responses as multiple answers were possible for this question*.

*“It's not a priority in the country because people feel that there are other issues that are more important like HIV and AIDS and other diseases like malaria. You find that there's more support for those as opposed to nutrition research.” Male, 45 years, Public sector, Zimbabwe*

*“It is those government organisations which allow funds to be allocated to specific sections of health care. That is where, I think, we have failed….it is essential for government to realise that a malnourished child and a malnourished adult will eventually cost the state a larger sum of money!” Female, 50 years, Academic, South Africa*

In addition, 9% of responses indicated that nutrition research was often perceived to be driven by the interest and agenda of international donors or policymakers. The latter were perceived to prefer direct results through short-term research in order to have immediate return on investment and to assist in quick decision-making. Moreover, 9% of the responses provided by interviewees indicated that the absence of a national coordinating body for nutrition at governmental level hampered the ability to move nutrition research higher up the political agenda.

Suggestions from nutrition researchers to prioritise nutrition research included more financial support (19% of responses) and attention from the government (12% of responses), and investment in capacity development for research (13% of responses). Furthermore, the answers showed the need for a research agenda implemented at national level (18% of responses). Nutrition researchers recognised the importance of national priorities to attract attention or funding, stressing that actual implementation of the agenda and communicating it as the official government's list of priorities was fundamental. Various research institutions were reported to have their own individual research agendas, but many researchers expressed the need for a joint national research agenda.

*“Ooh, I think the coordination is missing. I think like the university have their own agenda of nutrition, and probably TFNC [Tanzania Food and Nutrition Centre] have their own agenda and there is not a governing body coordinating research. We have the national [body] like COSTECH [Tanzania Commission for Science and Technology] coordinating the policies of research but, for nutrition [research] I think we should have a body coordinating nutrition, research priorities, and how to involve stakeholders [in] all that.” Female, 43 years, Public sector, Tanzania*

Nutrition researchers also perceived their own role as critical in prioritising nutrition research in the country. Several researchers indicated the need for advocacy to attract political attention (11% of responses). The establishment of a coordinating body or centre for nutrition research was seen as crucial in generating political interest by some (7% of responses).

*“I think there is a need for nutritionists to be together so that they can push for an agenda. You see if nutritionists have a body that can push for an agenda it is going to be very easy but, if you are disintegrated everywhere and not working together it is really hard to push for an agenda.” Female, 25 years, UN International organisation, Kenya*

### The utilisation of nutrition research findings

Over one-third (37%) of the nutrition researchers interviewed indicated that research findings were not utilised to inform policy (Table 3), whereas 22% of participants reported that research findings were infrequently utilised. Another 21% believed that only the findings from a few studies were used for policymaking, mainly those from international organisations. Most political support was believed to exist for fortification and supplementation programmes, such as iodine, in many cases based on studies acknowledged by international organisations (data not tabulated). The limited interaction between nutrition researchers and policymakers was considered a key factor in explaining the poor translation of local research evidence into policy (41% of responses). Local research findings were often not used for policy in this regard because studies were considered small-scale, superficial or descriptive, which was attributed mainly to low research capacity (16% of responses). In addition, inadequate understanding of nutrition issues by policymakers was believed to cause the low demand for nutrition research for policy making (7% of responses).

**Table 3:** Linkages of policy and nutrition research as perceived by nutrition researchers in sub-Saharan Africa.

Are research findings used for policymaking?	n = 133^a^	%
No	49	37
Infrequently	30	22
Depends on the research	28	21
Yes	23	17

Reasons for no integration of nutrition research results into policy	n = 140^b^	%
No interaction between researchers and policymakers	57	41
Data is not good enough (superficial, descriptive, small-scale)	22	16
Funding constraints for implementation of results	12	9
Absence of formal links/forum/unit or isolation of nutrition researchers	11	8
Lack of understanding of research language/nutrition by policymakers	10	7
Research done for personal or donor interest and findings are not shared	9	6
Others	8	6

Suggestions for integration of nutrition research results into policy	n = 125^b^	%
Better interaction researchers and decision makers	30	24
Good research, i.e. more specific, problem-driven, more capacity to do research	17	14
Decision makers must be more active in research process/coordinate/bring stakeholders together	15	12
Need for a national forum, platform or council	13	10
Nutrition researchers represented at national level (more influence)	12	10
Data dissemination and validation through publication, seminars, policy briefs	11	9
Support government, i.e. funding schemes	11	9
Advocacy by nutrition research towards policymakers	10	8
Others	6	5

a *Number of researchers as only one answer was possible for this question.*

b *Total number of responses as multiple answers were possible for this question.*

Suggestions to enhance the link between researchers and policymakers were varied and included greater active involvement of decision makers in the research process regarding priority setting, data validation and coordination (12% of responses), establishing formal links between policy makers and researchers such as a platform or forum (10% of responses) and the representation of nutrition researchers at governmental level (10% of responses). This would contribute to better dissemination and validation of findings, which was also regarded as fundamental to increase research impact (9% of responses).

*“Researchers should be given a platform to air the research results, not just in scientific gatherings or conferences. If there are platforms where nutrition researchers and policymakers come together and the researchers break down their research in simple language for decision makers to understand what it means.” Female, 29 years, Academic, Ghana*

Current nutrition research was perceived as constrained by low local capacity and context and often conducted for the sake of personal or donor interest instead of for the benefit of the country. Hence, nutrition researchers highlighted the need to enhance the relevance of current research as regards national needs (14% of responses) and uptake for policy-making. The need to render research more problem-driven, with a stronger focus on prevention and a desire for research that benefits all parties was expressed.

*“The funders may be interested in a particular area, and sometimes it diverts you from what is very important to what the researcher wants. So it may be important for them as funders but it may not be important to you as a country. Of course the researcher will go to where the resources are but we want to get to a situation where both the researcher or nation and funder benefit.” Female, 45 years, Public sector, Uganda*

### Agenda-setting process for the research institution and funding

The primacy of individual researchers or research institutions in determining the institutional research agenda was reported in 50% of all responses (Table 4). Donors and international partners were considered to have a major influence in determining research priorities, albeit secondary to researchers themselves (28% of responses). Governments on the other hand, were seen as needing to be more involved in the agenda-setting process (31% of responses) than they currently are (18% of responses). A few nutrition researchers suggested that the local community could be an actor to set priorities for research.

**Table 4:** Responses related to agenda-setting of nutrition research as perceived by nutrition researchers in sub-Saharan Africa.

Actors that have a role in the agenda-setting process for your institution	n = 202^a^	%
Researchers or research institution	101	50
Donors or partners	56	28
Government or public institutions	37	18
Community	4	2
Others	4	2

Actors that should have a role in the agenda-setting process for your institution	n = 277^a^	%
Researchers or research institution	132	48
Government or public institutions	86	31
Donors or partners	33	12
Community	17	6
Others	9	3

a *Total number of responses as multiple answers were possible for this question.*

International donors were seen as the main funders of nutrition research (43% of responses). Participants reported that research was funded by these donors according to their priorities and often without consulting with African researchers or research institutions. The financial role of the government was perceived to be relatively limited (25% of responses, Table 5).

**Table 5:** Drivers of nutrition research as perceived by nutrition researchers in sub-Saharan Africa.

Current main funders of nutrition research	n = 236^a^	%
International organisations/NGOs	102	43
National Governments	59	25
National donors	23	10
University	19	8
Industry	12	5
No funding	11	5
Others	10	4

Influence of donors	n = 107^a^	%
Donors funding in line with their interests	70	65
Partnership between donors and researchers	14	13
No funds, research is financed from researcher's salary	12	11
Funders support agenda of researchers/institution	7	7
Others	4	4

Factors that determine the agenda-setting	n = 229^a^	%
Observation and experience of problems and needs	86	38
Funds available	82	36
Available human resources, i.e. capacity, lack of researchers	20	9
Limited equipment or facilities	17	7
Interest/motivation	11	5
Others	13	6

a *Total number of responses as multiple answers were possible for this question.*

*“In institutions like ours, we can't do research when there is no money, so the topics are always oriented towards the financed areas. But are the funded areas priority and beneficial for the population? Therefore we must be financially autonomous, and it is there that the government has its role to play. Only then can we be sure to address appropriately the problems of our country.” Female, 35 years, Academic, Benin*

A critical factor to determine the existing research agendas was the nutrition needs and problems, as reported in 38% of all responses (Table 5). However, more researchers pointed towards constraining factors as being influential in shaping the agenda, including available funding, human resources and resources for infrastructure (in 36%, 9% and 7% of all responses respectively). Most of the time it was seen as a balancing act between the identified needs on the one hand and the limited resources on the other that determined the research agenda of an institution.

### Improve the nutrition research capacity

Human resource capacity building was perceived to be the most urgent priority to advance nutrition research in sub-Saharan Africa (24% of responses, Table 6). Capacity building refers here to the volume of the research community, research skills (e.g. data analysis, research methods, proposal writing, English) and those that are specifically related to nutrition studies (including dietary assessment and laboratory analysis techniques), and the establishment of a specific education programme for nutrition research. It was envisaged that through this, research capacity and the voice of nutrition researchers would be fostered.

**Table 6:** Suggestions to improve nutrition research by nutrition researchers in sub-Saharan Africa.

Suggestions to improve nutrition research	n = 402^a^	%
Human resource capacity building, i.e. skills, higher education, more staff	95	24
Better equipment or infrastructure, i.e. lab equipment, internet, roads, etc.	65	16
Improved financial support in general	59	15
Collaboration/multidisciplinary research	54	13
Supportive policy context, i.e. priority/agenda/research body	42	10
Improve implementation/validation/inventory of results/forum to disseminate	21	5
Other research methods/topics, i.e. problem based, preventive, less curative	20	5
Improved communication opportunities, i.e. skills and meetings	15	4
Institutional/supportive research framework, i.e. mandate, time, etc.	14	3
Action by nutrition researchers, i.e. more advocacy or more interest	10	2
Others	7	2

a *Total number of responses as multiple answers were possible for this question.*

*“I think lack of nutritionists, dieticians. I think we are lacking because if we have this technical [expertise] in the country, we can all speak with the same voice. We can voice our concerns about the lack of information and need for research.” Female, 45 years, Public sector, Namibia*

Among the suggestions made, the quality of equipment and infrastructure (16% of responses) and better financial support (15% of responses) were mentioned most often. A political context conducive to nutrition research was perceived as imperative and included attention of government, national priority setting and a coordinating nutrition body at national level. Furthermore, collaboration was highly recommended within Africa because nutrition researchers were often perceived to be isolated, competing with each other or conducting overlapping research. Also international collaboration was rated highly by almost all researchers (99% of all interviewed), primarily for the benefits of exchanging knowledge and experience (35% of responses, data not tabulated). In addition, nutrition researchers proposed interdisciplinary collaboration as a necessary means to ensure a comprehensive approach to nutrition problems.

*“Create better teams in the university, teams who trust one another. I think that is the great problem at the university, it is much easier to work with the European Union [European based research groups] than with the colleagues next to him. The competitive atmosphere is just a result of our system of subsidy” Female, 50 years, Academic, South-Africa*

*“I think we need a bigger forum that brings all nutrition people working in Africa to come together. Nutrition is wide so that once we meet, people who work in different areas can discuss issues and then we can form a common agenda of what to be done in individual countries.” Female, 52 years, Research Institute, Kenya*

## Discussion

This article described the operating environment of nutrition research in sub-Saharan Africa from the perspective of African nutrition researchers and identified several key issues.

### Prioritising and re-orientating funding

The majority of researchers perceived the profile of nutrition research in their country as rather weak, mainly due to the lack of interest and support of the government. The lack of attention was in part attributed to governments' limited understanding of the benefits of adequate nutrition for development, confirming previous reports [5], [22]. Moreover, the researchers indicated that the current political environment for nutrition research tended to be reactive and directed towards emergency situations. Effective emergency responses were certainly considered indispensable, however these short-term solutions alone will not enable communities to become self-sufficient and food secure [14]. This argues for a long-term and proactive plan for nutrition research to be designed, in which emphasis should be placed more on prevention than on treatment. Moreover it was found that nutrition was often considered as subsidiary to other public health problems, such as infectious diseases and therefore received insufficient attention from the government. Policies neglecting nutrition and agriculture in favour of other investments were prevailing in some countries [2]. The triple burden of disease that sub-Saharan Africa faces requires a reduction of infectious diseases and undernutrition and control or prevention of non-communicable diseases. Therefore, the challenge remains to design appropriate, multifaceted, and multi-sectoral programmes that address under- and over nutrition jointly and holistically [23] apart from the central role of nutrition-specific interventions [9], [24].

Complementary to this need for re-orientating priorities, researchers also expressed governments' accountability in allocating more finances to nutrition research. One of the major problems as reported by the nutrition researchers interviewed for this study was the lack of financial support and the distortion of funds towards other priorities. Moreover international donor organisations were seen as main funders of nutrition research in Africa and researchers expressed concern that their research ideas should be tuned to the interests of the funders. A considerable distortion of research grants was previously observed, with the majority dedicated towards the areas with the least potential impact [25]. Funding areas such as food aid and supply-led technical assistance dominate whereas capacity investments and solution-oriented research are lacking [26], which calls for action in the donor community. Various countries where health or health-related research is non-existent are overlooked by funders, who believe that they can only invest where there is sufficient existing capacity to absorb resources [27]. In addition, shaping a national nutrition agenda was perceived to be crucial to integrate nutrition research in the development agenda of sub-Saharan African countries, to redirect funding and have less fragmented research. A national nutrition agenda is effective to ensure awareness of the country priorities, and thus would help to reflect them more in international donor funding [22]. The role of governments is critical in this regard, since it is the most powerful stakeholder in building ownership of a nutrition strategy at a national level [9]. Very few nutrition researchers considered local communities as key participating actors in shaping the agenda. This was not in line with previous reports [28] that have documented the role of local community engagement in priority setting for health research.

### Improved interaction and problem-driven research

Evidence and baseline data on the nutritional situation in a country were seen as crucial to creating political will, getting nutrition on the development agenda, as well as enabling evidence-based decision-making and organising effective interventions. Pelletier *et al.* [29] pointed out that among the most influential factors to raise the profile of nutrition research are providing clear evidence for the size and urgency of the problem and framing the problem in a way that has political resonance. Nevertheless this study revealed that according to nutrition researchers the findings of nutrition research have not been fully exploited to date in policy, practice or academic publications due to several constraints.

Firstly, nutrition researchers expressed additional concerns regarding the communication barrier between researchers and policymakers, which was viewed as preventing evidence from reaching the political level. Our results confirm that nutrition researchers felt the need to lobby for political awareness and put evidence for the current nutrition situation on the table. Despite the importance of dissemination, networking between researchers and policymakers is essential in a way that promotes continuous dialogue to strengthen the research contribution to policy and involvement of policy throughout the research process [30], [31]. Respondents in this study re-iterated this idea by suggesting a coordinating body at governmental level, which could serve as a major support for the involvement of researchers in policymaking and improved interaction between policy and research. However, Benson previously reported how various national coordinating bodies in a few sub-Saharan African countries have been ineffective to date [5].

Secondly, the current evidence base of nutrition research in Africa is largely descriptive and falls short of providing convincing data for policymakers to initiate national interventions or to trigger the investment for appropriate nutrition interventions to research priorities that are tailored to the African context [12], [26]. The nutrition researchers in our study argued that research needs to be more problem-driven and explained that this has been constrained by limited research capacity. It has been reported that adequate local research capacity development is key to ensure the use of evidence by local policymakers [31].

### Building capacity

Although some countries have national action plans on nutrition [32], they rarely include strategies to build capacity for research. Strengthening research capacity is critical to enable more policy- and programme- relevant nutrition research and to respond to local community nutrition health concerns [26], [33]. Moreover improved capacity was considered critical in building a stronger voice to advocate for political attention, as well as a positive incentive to avoid ‘brain drain’. Some nutrition researchers argued for the establishment of a higher education programme for nutrition research for improving skills and volume. An initiative to establish a Nutrition Research and Training programme in West Africa is under consideration [34]. However, it is clear that more efforts are needed to provide training programmes at all levels and need to be directed towards the attainment of specific nutrition research skills, as suggested by the interviewees. Concerted action by governments, international agencies, donor organisations and other stakeholders, including the private sector is important in fostering regional capacity building initiatives [34]. Recently, a major international initiative was launched [9] to bring nutrition to the fore. The Scaling Up Nutrition (SUN) movement will hopefully create a momentum for nutrition research in Africa to be considered in national nutrition strategies and investment in nutrition research in general.

### Enhance cross-African collaboration

Collaboration, in particular within Africa is another way to pool resources and to maximise the use of knowledge on nutrition in the continent to attract further funding. Cross-African linkages in research are limited and a considerable share of publications concerning Africa are associated with institutions that are situated outside of Africa [35]. Cross-African collaboration and increase of resource capacity were expected to create more African ownership for nutrition research. The findings of this study indicated that supporting collaborations could be attained by boosting research visibility and awareness, stimulating networks for knowledge exchange, building capacity, alleviating isolation and providing funding schemes for research carried by partners from various African countries. Notwithstanding, North-South collaboration was also perceived as indispensable by researchers, however the organisation of partnership programmes must be re-oriented to focus on capacity-building and include measures to avoid ‘brain drain’.

The present study provided a comprehensive overview of cross-cutting issues regarding the research environment of nutrition research from the perspective of sub-Saharan African researchers, from a wide variety of contexts and countries. With regard to the importance of nutrition research for sub-Saharan Africa, it contributes to the understanding of future requirements to enable nutrition research capacity building. Although we aimed to collect data from all the countries in sub-Saharan Africa, 12 countries did not provide data. Several of these countries are known to have lower research output because of political, geographical or historical reasons [27]. Our results might not apply to these countries and primarily relate to countries where a considerable amount of research is already being conducted. Although this study provides an overview of the most important themes emerging from nutrition researchers in sub-Saharan Africa, it did not explore the underlying reasons for the answers.

The sample of people interviewed covered a heterogeneous group, since people involved in nutrition research from academic, public sectors and other local/international organisations were recruited. Despite this however, we observed a significant consistency in the responses, which indicates the robustness of our results. We acknowledge that actions to improve the operating environment for African nutrition researchers will require engagement by various stakeholders, including those outside of the research community. Within SUNRAY, a stakeholder analysis was carried out in this regard. The findings will be presented elsewhere.

In conclusion, nutrition research in sub-Saharan Africa is at a crossroads. A substantial amount of effort is devoted to nutrition in the region. While national government and donors require high quality evidence to prioritise their actions in nutrition, African researchers highlighted a number of key barriers to achieve this. Apart from capacity development and actions to strengthen human resources, priority setting and the development of a local research agenda based on priorities tailored to the African context needs to be a key priority. In addition, nutrition researchers stated that they wanted a stronger voice to advocate for political commitment in nutrition research in their country, supported by a coordinating body for nutrition research to increase the interaction between researchers and policymakers and consequently facilitate validation of research findings. Investment in multidisciplinary and international collaboration, with cross-African linkages, may offer important avenues to support the research capacity in sub-Saharan Africa.

## References

[1] United Nations Standing Committee on Nutrition (UNSCN) (2010) Sixth report on the World Nutrition Situation: Progress in nutrition. Geneva, ACC/SCN.

[2] UNDP (2012) Africa Human Development Report: Towards a food secure future. New York: United Nations Development Programme.

[3] FAO (2003) The state of food insecurity in the world. Rome: Food and Agriculture organisation of the United Nations.

[4] World Bank (2006) Repositioning Nutrition as Central to Development: A Strategy for Large Scale Action. Washington, D.C.: World Bank.

[5] Benson T (2008) Improving Nutrition as a Development Priority Addressing Undernutrition in National Policy Processes in Sub-Saharan Africa. Washington, D.C.: International Food Policy Research Institute.

[6] Kelly T, Yang W, Chen CS, Reynolds K, He J (2008) Global burden of obesity in 2005 and projections to 2030. Int J Obes 32: 1431–1437. 10.1038/ijo.2008.102 18607383

[7] World Health Organization (2004) Global Strategy on Diet, Physical Activity and Health. Geneva: World Health Organization.

[8] World Health Organization (2008) 2008–2013 Action Plan for the Global Strategy for the Prevention and Control of non-communicable diseases. Geneva: World Health Organization.

[9] Scaling-Up nutrition Roadmap task team (2010) A Road Map for Scaling-Up Nutrition (SUN). New York: UN. Available: http://unscn.org/files/Activities/SUN/SUN_Road_Map_english.pdf. Accessed 2012 Jun 13.

[10] Bill and Melinda Gates Foundation (2011) Nutrition Strategy Overview. Available: http://www.gatesfoundation.org/global-health/Documents/nutrition-strategy.pdf. Accessed 2012 Jun 24.

[11] GAIN (2012) GAIN's Nutrition Program. Available: http://www.gainhealth.org/programs. Accessed 2012 Jun 24.

[12] Department for International Development (2010) The neglected crisis of undernutrition: DFID's Strategy. UK: DFID. Available: http://collections.europarchive.org/tna/20100423085705/http:/dfid.gov.uk/Documents/publications/nutrition-strategy.pdf. Accessed 2012 Jun 24.

[13] G8 Camp David Summit (2012) Fact Sheet: G-8 Action on Food Security and Nutrition.

[14] UNDP UNECA, AU AfDB (2011) Assessing Progress in Africa toward the Millennium Development Goals. MDG Report 2011. New York: UNDP.

[15] Stevens GA, Finucane MM, Paciorek CJ, Flaxman SR, White RA, et al (2012) Trends in mild, moderate, and severe stunting and underweight, and progress towards MDG 1 in 141 developing countries: a systematic analysis of population representative data. The Lancet 380: 824–834. 10.1016/S0140-6736(12)60647-3 PMC3443900 22770478

[16] Aaron GJ, Wilson SE, Brown K (2010) Bibliographic analysis of scientifc research on selected topics in public health nutrition in West Africa: Review of articles published from 1998 to 2008. Global Public Health 5: S42–S57. 21113830 10.1080/17441692.2010.526128

[17] Sawyerr A (2004) African Universities and the Challenge of Research Capacity Development. JHEA/RESA 2: 211–240.

[18] World Bank (2010) World Development Indicators. Washington, D.C.: World Bank. Available: http://data.worldbank.org/sites/default/files/wdi-final.pdf. Accessed 2012 Mar 8.

[19] Borregaard N (2002) Drivers of Knowledge Production: Research Partnerships for Sustainable Development. London: International Institute for Environment and Development (IIED).

[20] Beattie P, Renshaw M, Davies CS (1999) Strengthening Health Research in the Developing World. Malaria Research Capacity in Africa. London: Wellcome Trust for the Multilateral Initiative on Malaria.

[21] Weber RP (1990) Basic Content Analysis. Newbury Park, CA: Sage.

[22] Bryce J, Coitinho D, Darnton-Hill I, Pelletier D, Pinstrup-Andersen P (2008) Maternal and child undernutrition: effective action at national level. The Lancet 371: 510–526. 10.1016/S0140-6736(07)61694-8 18206224

[23] Vorster H, Bourne L, Venter C, Oosthuizen W (1999) Contribution of Nutrition to the Health Transition in Developing Countries: A Framework for Research and Intervention. Nutrition Reviews 57: 341–349. 10628185 10.1111/j.1753-4887.1999.tb06911.x

[24] Meerman J (2008) Making Nutrition a National Priority: Review of Policy Processes in Developing Countries and a case-study of Malawi. Rome: FAO (Nutrition Requirements and Assessment Service).

[25] Leroy JL, Habicht J, Pelto G, Bertozzi SM (2007) Current Priorities in Health Research Funding and Lack of Impact on the Number of Child Deaths per Year. Am J Public Health 97: 219–223. 17194855 10.2105/AJPH.2005.083287 PMC1781402

[26] Morris SS, Cogill B, Uauy R (2008) Effective international action against undernutrition: why has it proven so difficult and what can be done to accelerate progress? The Lancet 371: 608–621. 10.1016/S0140-6736(07)61695-X 18206225

[27] McKee M, Stuckler D, Basu S (2012) Where There Is No Health Research: What Can Be Done to Fill the Global Gaps in Health Research? PLoS Med 9: e1001209. 22545025 10.1371/journal.pmed.1001209 PMC3335864

[28] Batista R, Berger M, Devlin M, de Haan S, Djamalova M, et al.. (2006) Can communities influence national health research agendas? COHRED Record Paper 3. Geneva: Council on health research for Development (COHRED).

[29] Pelletier DL, Frongillo EA, Gervais S, Hoey L, Menon P, et al (2012) Nutrition agenda setting, policy formulation and implementation: lessons from the Mainstreaming Nutrition Initiative. Health Policy Plan 27: 19–31. 21292709 10.1093/heapol/czr011

[30] Brownson RC, Royer C, Ewing R, McBride T (2006) Researchers and policymakers- travellers in parallel universes. Am J Prev Med 30: 164–172. 16459216 10.1016/j.amepre.2005.10.004

[31] Vincent R (2006) Communicating health research: how should evidence affect policy and practice? Findings No 5: 6.

[32] World Health Organization (2010) A Review of Nutrition Policies. Draft Report 20 December 2010. Available: http://www.who.int/nutrition/EB128_18_Backgroundpaper1_A_review_of_nutritionpolicies.pdf. Accessed 2012 Jun 6.

[33] Williams JR, Schatz EJ, Clark BD, Collinson MA, Clark SJ, et al (2010) Improving public health training and research capacity in Africa: a replicable model for linking training to health and socio-demographic surveillance data. Glob Health Action doi:10.3402/gha.v3i0.5287 10.3402/gha.v3i0.5287 PMC2932506 20824101

[34] Brown KH, McLachlan M, Cardosa P, Tchibindat F, Baker SK (2010) Strengthening public health nutrition research and training capacities in West Africa: Report of a planning workshop convened in Dakar, Senegal, 26–28 March 2009. Glob Public Health 5: S1–19. 21113828 10.1080/17441692.2010.526126

[35] Adams J, King C, Hook D (2010) Global Research Report. Africa. Leeds, UK: Evidence, a Thomson Reuters business.

